# SCAT8/miR-125b-5p axis triggers malignant progression of nasopharyngeal carcinoma through SCARB1

**DOI:** 10.1186/s12860-023-00477-2

**Published:** 2023-04-03

**Authors:** Chunmao Jiang, Dandan Feng, Yu Zhang, Kun Yang, Xiaotong Hu, Qian Xie

**Affiliations:** 1 Department of Health Management, Daping Hospital, Army Medical University, Chongqing, 400010 China; 2 Department of Otolaryngology Head and Neck Surgery, Daping Hospital, Army Medical University, Chongqing, 400010 China; 3 Department of Otolaryngology Head and Neck Surgery, the First Affiliated Hospital of Chongqing Medical University, Chongqing, 400010 China; 4 Department of Health Management Center, the Second Affiliated Hospital of Chongqing Medical University, Chongqing, 400010 China

**Keywords:** Nasopharyngeal carcinoma, ceRNA, SCARB1, Cell proliferation, Cell migration

## Abstract

Nasopharyngeal carcinoma is a tumor with high malignancy and poor prognosis, which severely affects the health of the patients. LncRNAs and microRNAs are crucial for the occurrence and development of nasopharyngeal carcinoma, which regulate the progression of nasopharyngeal carcinoma through the ceRNA network. SCARB1 plays an essential role in nasopharyngeal carcinoma. However, the mechanism underlying the regulation of SCARB1 in nasopharyngeal carcinoma through non-coding RNAs remains unclear. Our findings indicated that the SCAT8/miR-125b-5p axis promoted the malignant progression of nasopharyngeal carcinoma by driving the expression of SCARB1. Mechanistically, the expression of SCARB1 could be regulated by the lncRNA, SCAT8 and the microRNA, miR-125b-5p. Moreover, as a ceRNA of miR-125b-5p, SCAT8 can not only regulate the expression of SCARB1, but also regulate the malignant progression of nasopharyngeal carcinoma. Notably, our results reveal a novel ceRNA regulatory network in nasopharyngeal carcinoma, which could serve as a potential target for the diagnosis and treatment of nasopharyngeal carcinoma.

**Supplementary Information:**

The online version contains supplementary material available at 10.1186/s12860-023-00477-2.

## Introduction

Nasopharyngeal carcinoma occurs in the nasopharynx, nasopharyngeal cavity, and upper throat, and is common across Southeast Asia and Africa [28]. The position of the nasopharynx in the center of the head, close to the base of the skull, with significant blood vessels and nerves passing nearby, makes it highly susceptible to cervical lymph node metastases [34]. Early-stage patients have a cure rate of more than 80%, but nasopharyngeal carcinoma in the early stage is difficult to detect due to the location of the nasopharynx [30]. More than 70% of the patients see a doctor regularly for common symptoms like neck lumps and nasal congestion, which are similar to those of nasopharyngeal cancer [33]. In the early stages, radiotherapy alone shows excellent results but patients with an advanced or recurrent disease may need a combination of chemotherapy and surgery [14]. After regular treatment, the average five-year survival rate is nearly 60% and more than 30% for advanced patients [37]. Scientists worldwide have been searching for effective treatment strategies to reduce the recurrence rate of nasopharyngeal carcinoma and improve patient survival.

Non-coding RNA (ncRNA) refers to RNA without protein-coding ability, and mainly includes circular RNA (circRNA), long non-coding RNA (lncRNA), and micro RNA (miRNA), among other types [12, 32]. Because ncRNAs are closely related to human diseases, their study can provide new ideas and theoretical foundations for the occurrence, development, and treatment of several disorders [3]. miRNA is a kind of endogenous, short-sequence, non-coding single-stranded sRNA, generally consisting of 18–25 nucleotides [21]. The mechanism of miRNA action is mainly divided into the two following categories: one, whereby miRNA closely binds to the ORF region of the mRNA, forming a double-stranded structure for its degradation; the other, whereby the miRNA loosely binds to the 3’UTR region of the mRNA, thus affecting its post-transcriptional translation [20, 31, 43]. miRNA mainly acts on the target gene by cutting off the target gene mRNA molecule, preventing the translation, and binding to and inhibiting the target gene, thereby playing a role in cellular differentiation, apoptosis, proliferation, metabolism, homeostasis, signaling transduction, auto-immunity, inflammatory responses, tumor intervention, and various other physiological and pathological processes [8, 9,16]. miRNA inhibits tumor cell growth, is downregulated in most tumor tissues, and affects tumor cell proliferation and invasion through the regulation of target proteins [44]. miRNA plays a critical role in tumor cell metastasis, which provides new ideas for the diagnosis and treatment of these patients [2, 4]. Specific miRNAs can reflect the tissue origin of tumors and molecular typing can be conducted to predict the prognosis and estimate the treatment sensitivity of patients, and even directly participate in the treatment [19, 48]. microRNA-125b is differentially expressed across tumor types and can regulate cell proliferation and apoptosis. miR-125b-5p shows an inhibitory effect on the invasion and migration of ovarian cancer cells [26, 42]. As a promising new plasma biomarker, hsa-miR-125b-5p can be employed as one of the biomarkers of colorectal cancer because its expression is closely associated with colorectal cancer [10, 18]. Therefore, the hsa-miR-125b-5p probe is commonly used for detecting colorectal cancer. However, in nasopharyngeal carcinoma, the research and application of hsa-miR-125b-5p are elusive.

LncRNA is an RNA molecule with a transcript length exceeding 200 nucleotides [41]. Although lncRNAs do not encode a protein, they often function as RNA to regulate protein coding at different levels including transcriptional, post-transcriptional, and epigenetic regulation [5]. LncRNAs can interact with DNA, mRNA, and proteins [39]. These include direct interaction with the promoter region of the coding genes, mediation of histone modification, chromatin remodeling, regulation of variable mRNA precursor splicing, or formation of endogenous siRNA molecules [6]. Abnormal expression of lncRNAs in various cancer samples including nasopharyngeal carcinoma is relevant for regulating the proliferation, invasion, migration, and apoptosis of tumor cells [11, 40]. However, studies on lncRNAs in nasopharyngeal carcinoma are scarce, and the existing reports mainly focus on a single lncRNA. Thereby, an in-depth study of the mechanism of lncRNA action in nasopharyngeal carcinoma is of great significance for its occurrence and development, which is conducive to early diagnosis, targeted therapy as well as improved prognosis of these patients. Recently S-phase cancer-associated transcript 8 (SCAT8), a key tumor marker of S-phase lncRNA, has gained research traction [1]. However, studies focused primarily on the mechanism of SCAT8's action in tumors are lacking.

Scavenger Receptor Class B type I (SR-BI), encoded by Scavenger Receptor Class B Member 1 (SCARB1), is the plasma membrane receptor of high-density lipoprotein (HDL) cholesterol, mediating the transfer between cholesterol and HDL [24]. The proliferation and invasion of tumor cells, as well as the enhanced ability to adapt to the tumor microenvironment, are often closely related to the abnormally active cholesterol metabolism [15, 45]. Therapeutic strategies targeting cholesterol synthesis and its related proteins may shed new insight into the treatment of tumors [46]. Studies have confirmed that SR-BI causes proliferation, migration, and enhancement of tumor cell growth in xenograft tumor models of breast cancer [17]. In some rare studies on SR-BI in nasopharyngeal carcinoma, increased expression of SR-BI was found to be related to enhanced cell migration ability [47]. CircRNA-scavenger receptor class B member 1 (Circ-SCARB1) is high in 30 pairs of renal cell carcinoma tissue cell lines [27]. Down-regulated expression of circ-SCARB1 can restrain cell proliferation, migration, and invasion, and induce cellular apoptosis [25]. However, studies of SCARB1 in lncRNA are scarce, and the regulatory mechanism underlying its receipt of ncRNA in nasopharyngeal carcinoma remains elusive. Therefore, the regulation of SCARB1 in nasopharyngeal carcinoma was studied herein.

In this study, we found that SCAT8 served as a ceRNA for miR-125b-5p to control SCARB1 expression in nasopharyngeal carcinoma. First, we identified the regulatory functions of SCAT8, miR-125b-5p, and SCARB1 in the malignant progression of nasopharyngeal carcinoma. Next, we predicted and verified SCAT8’s binding to miR-125b-5p and molecular regulation of SCARB1. Finally, we found that the SCAT8/miR-125b-5p axis could regulate the proliferation and migration of nasopharyngeal carcinoma cells through SCARB1. This study lays a theoretical foundation for the development of targeted therapy and improvement in the prognosis of patients with nasopharyngeal carcinoma.

## Methods

### Data collection

The mRNA, lncRNA, and miRNA sequencing data of 58 nasopharyngeal carcinoma patients and their corresponding clinical information were downloaded from The Cancer Genome Atlas (TCGA) database for cluster and Cox regression analyses.

### Univariate Cox analysis

Univariate Cox analysis was performed to evaluate the overall survival rate and status of nasopharyngeal carcinoma patients in TCGA database. mRNA, lncRNA, and miRNA data of nasopharyngeal carcinoma patients were assessed based on a *p*-value < 0.05.

### Differential gene expression analysis

The data for differential expression analysis was normalized using the limma package. By comparing the differentially expressed genes between nasopharyngeal carcinoma tumor samples and paracancerous samples, mRNAs, lncRNAs, and miRNAs were analyzed based on the conditions, logFC ≥ 1 and *p*-value < 0.05.

### Gene enrichment analysis

The biological regulatory effects of differentially expressed genes for mRNAs were evaluated by gene enrichment and pathway analyses. The overlapping genes enriched in different signaling pathways were analyzed using the R package, clusterProfiler, following differential gene expression analysis. If the *p*-value < 0.05, the enrichment of genes in the corresponding pathways was regarded as significant.

### Cell culture

The nasopharyngeal cancer cell line, C666-1, was purchased from ATCC and grown in RPMI-1640 supplemented with 10% FBS, 100 μg/mL penicillin, and 100 μg/mL streptomycin. The cells were maintained in a humidified incubator with 5% CO_2_ at 37 ℃.

### Real-time quantitative polymerase chain reaction (RT-qPCR)

Total RNA was isolated from the C666-1 cell line following the kit protocol. Subsequently, the cDNA was synthesized by reverse transcription. RT-qPCR was performed following the manufacturer’s instructions. The levels of expression of 5 lncRNAs related to mRNA were determined by calculating the ratio of the transcripts in the samples. GAPDH was used as the endogenous control to calculate the relative expression. The primer sequences used for RT-qPCR are listed in Table 1.

**Table 1:** The primers in RT-qPCR

SCAT8	Forward:	5’-TGGTTGGGCTTGAAAGTGTA-3’
	Reverse:	5’-CCAGCTGGTTTTCAGTCTCC-3’
	miR-125b-5p Universal R-primer:	5’- TCCCTGAGACCCTAACTTGTGA-3’
SCARB1	Forward:	5’-TGCAGTGTTTCACCTTGCAT-3’
	Reverse:	5’-GCCTCGGAAAACAACTTCTG-3’

### Dual-luciferase reporter assay

PGL4.15-control luciferase was inserted into the vector and the wild-type (WT) and mutant (Mut) SCAT8 3' UTR regions were amplified by PCR. C666-1 cells were seeded into the 24-well plate. After cell adhesion, WT and Mut SCAT8 were transfected into C666-1 cells with Lipofectamine 3000 (Invitrogen). After transfection, cells were collected and the luciferase activity was detected on the Dual-Luciferase reporter assay system (Promega). The relative firefly luciferase activity was normalized against the renilla luciferase activity.

### Cell proliferation assay

The CCK-8 reagent was used to detect cell proliferation. Briefly, C666-1 cells were inoculated into 96-well plates at a density of 1000 cells per well. After 48 h of cell growth, 10 μL of the CCK8 reagent was added per well. Next, the cells were cultured at 37 ℃. After 3 h, the absorbance values of wells were detected at 450 nm using a microplate reader.

### Pearson's correlation analysis

The value of the Pearson correlation coefficient varies from—1 to 1 and it is used to detect the correlation between two variables. When the Pearson correlation coefficient is 1, it means that all data points of the variable are on a straight line and the variable Y increases with the increase in variable X. When the Pearson correlation coefficient is -1, it implies that all data points fall on a straight line. When the Pearson coefficient is 0, it indicates that the two variables have no linear relationship.

### Plotting the receiver operating characteristic curve (ROC)

The area under the curve (AUC) indicated the sensitivity of lncRNA for survival. The diagnostic value of the ceRNA axis in nasopharyngeal carcinoma was evaluated using the ROC.

### Transfection

Inhibitors and mimics of miRNAs were bought from Biomics (Nantong, China). The ASOs of lncRNA SCAT8 were purchased from RiboBio (Guangzhou, China). Inhibitors, mimics, and ASOs of miRNA were transfected into C666-1 cells using Lipofectamine 3000 (Invitrogen, US) following the manufacturer’s instructions. shRNA of SCARB1 was inserted in the PLKO.1 plasmid. The shRNA sequence was shSCARB1 5’-AUAAUCCGAACUUGUCCUUGAAGGG-3’.

### Transwell migration assay

Cell migration ability was detected by the Transwell migration assay. The Transwell chamber was placed into a 24-well plate and the starved cells were resuspended in a serum-free medium. Subsequently, 200 μL cell suspension per well was added into the chamber. The complete medium was added to the bottom of the chamber. After 12–24 h of cell culture, the inner and outer layers of the culture medium were discarded and cells were washed with PBS. Polyformaldehyde was used to fix cells. After 15 min of fixation, 0.2% crystal violet dye was used to stain the cells and PBS was used to wash the residual dye. A cotton swab was used to wipe-off non-migrated cells and images of migrated cells were captured.

### Construction of the lncRNA-miRNA-mRNA ceRNA network

The ceRNA network of lncRNA-miRNA-mRNA was constructed by predicting the correlations among lncRNAs, miRNAs, and mRNAs based on the ceRNA theory. miRNA was a key component of the ceRNA network, playing a central role in connecting lncRNAs and mRNAs. The network was visualized using the Cytoscape software.

### Statistical analysis

The experimental data are presented as mean ± standard deviation of three independent experiments. LSD-t test was used for statistical analysis between two groups and ANOVA was performed for comparison among multiple groups. When the *p*-value was less than 0.05, the results were considered statistically significant. GraphPad Prism software was used for the analysis and production of statistical charts and the Image J software was used for the grayscale analysis of protein blots. The Kaplan–Meier method was used for plotting the survival curve.

## Results

### Screening of lncRNAs, microRNAs, and mRNAs related to the progression of nasopharyngeal carcinoma

**Fig. 1:**
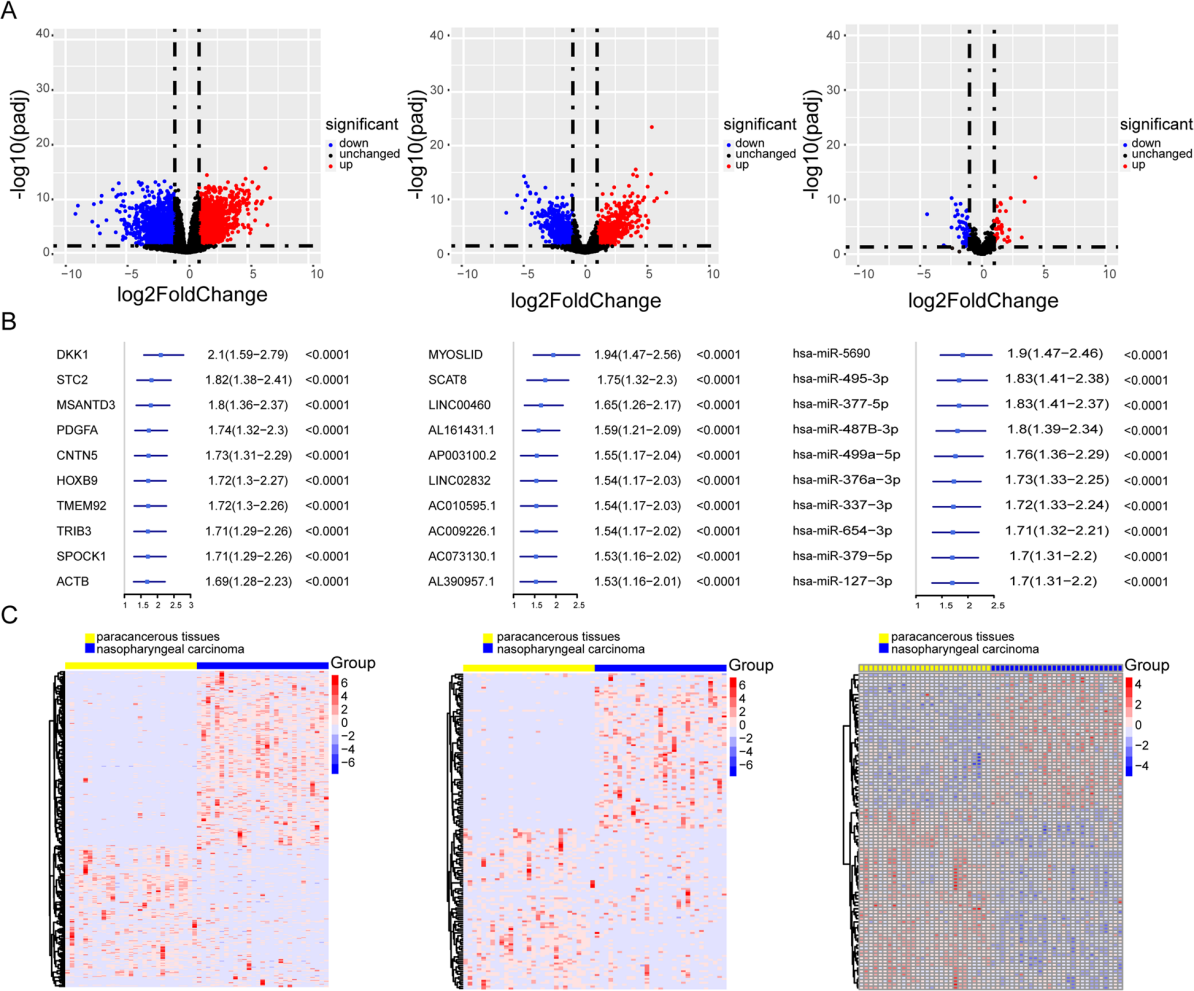
The results of univariate cox and differential expression analysis of mRNAs, lncRNAs and miRNAs from the TCGA-NPC database. Volcano plots of differential expression analysis of mRNAs, lncRNAs and miRNAs (**A**). The top ten results of between univariate cox and differential expression analysis in mRNAs, lncRNAs and miRNAs (**B**). Heatmaps of mRNAs, lncRNAs and miRNAs (**C**)

During tumor development, the expression of tumor markers changes significantly with tumorigenesis. Several markers are oncogenes or tumor suppressor genes, regulating the malignant progression of tumors. However, the role of the ceRNA axis comprising these genes in nasopharyngeal carcinoma has not been clarified. To assess its underlying mechanism, differential expression analysis, and univariate Cox analysis were performed based on the mRNAs, lncRNAs, and miRNAs in nasopharyngeal carcinoma tumor samples and paracancerous samples (Fig. 1A, B and Fig. S1A). The upregulated oncogenes and downregulated suppressor genes in nasopharyngeal carcinoma were extracted after obtaining the overlapping differentially expressed genes and survival-related genes, including 406 protein-coding mRNAs and 165 lncRNAs (Fig. 1C and Fig. S1B).

### Enrichment of tumor progression-related genes in nasopharyngeal carcinoma

**Fig. 2:**
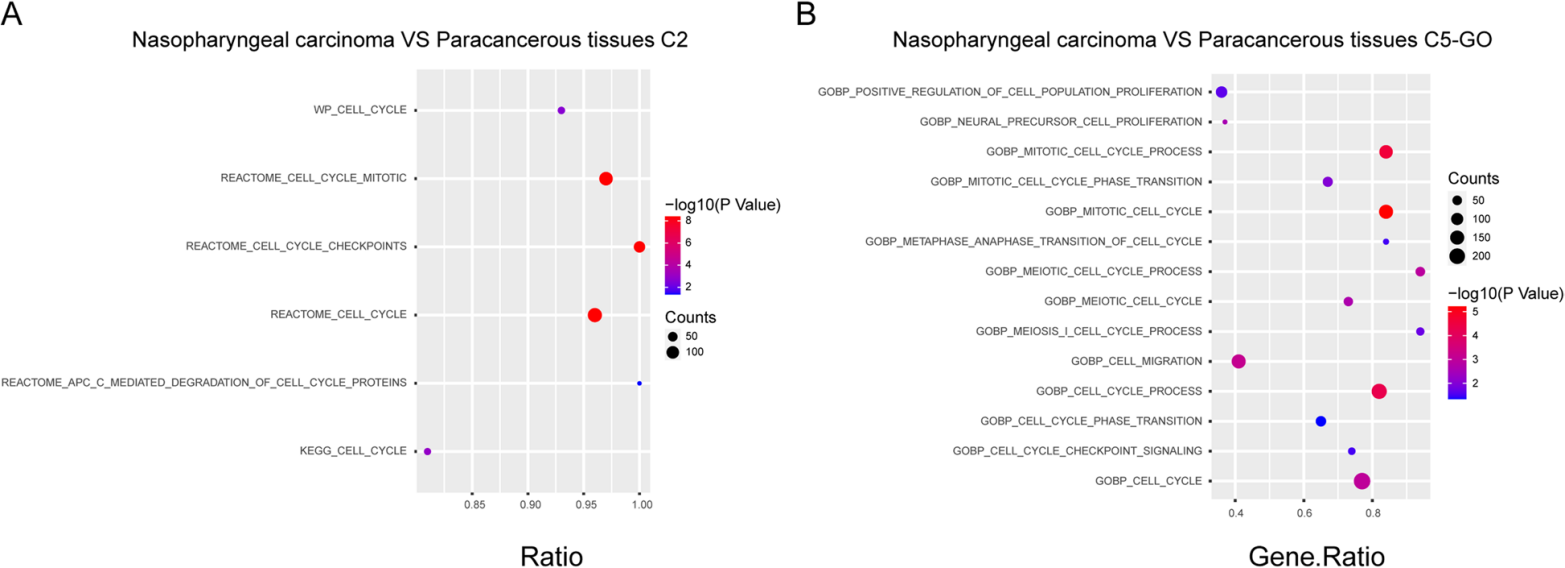
C5 GO fuctional and C2 pathway enrichment analysis of the diferentally exressed mRNAs. Bubble diagram of C2 pathway analysis of mRNAs (**A**). Bubble diagram of C5 GO analysis of mRNAs (**B**)

In order to further assess the importance of these genes in nasopharyngeal carcinoma tumors, GSEA was performed for evaluating mRNA enrichment. The enrichment of the C2 pathway indicated that the genes were chiefly overrepresented in cell cycle-related pathways (Fig. 2A and Fig. S2A). C5 GO enrichment indicated that the genes were also involved in cell cycle, cell proliferation, and cell migration (Fig. 2B and Fig. S2B).

### SCAT8/miR-125b-5p/SCARB1 may be an important ceRNA axis regulating nasopharyngeal carcinoma

**Fig. 3:**
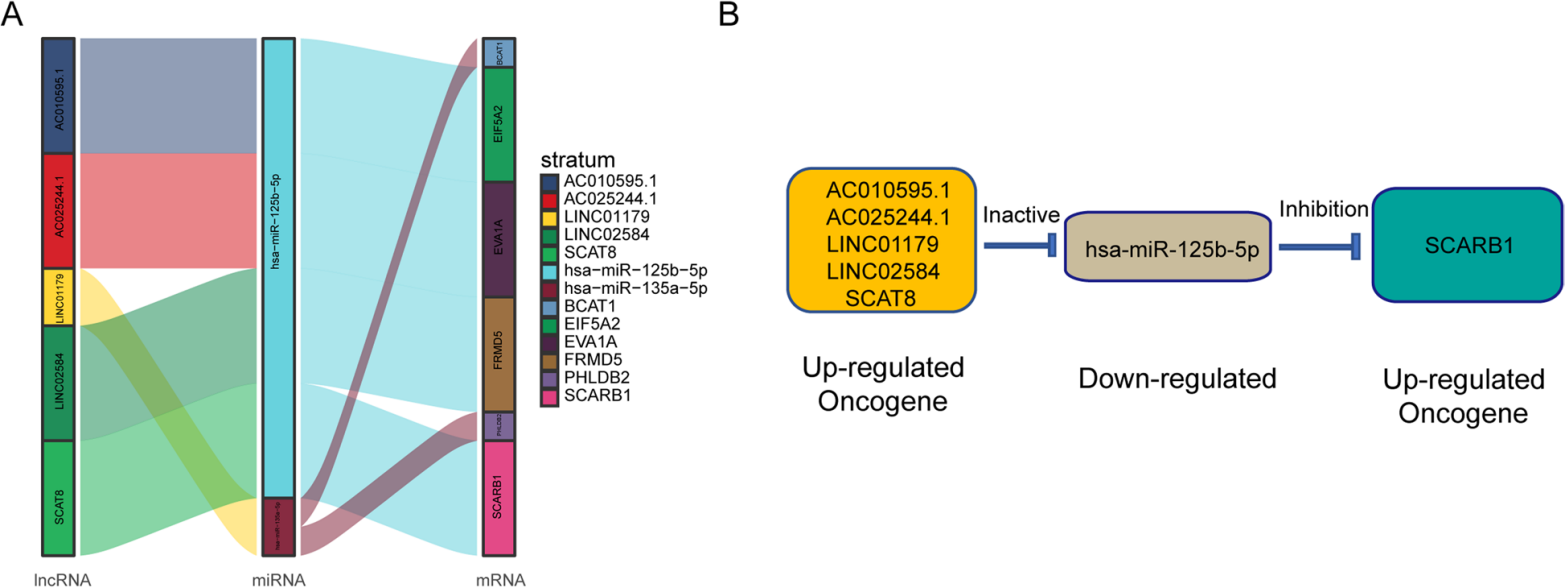
Construction of nasopharyngeal carcinoma related ceRNA network by integrated analysis. LncRNA-miRNA-mRNA regulatory axes extracted from this ceRNA network (**A**). The LncRNA-miRNA-mRNA pattern diagram (**B**)

According to the above results, the ceRNA network was constructed. It was found to regulate the malignant progression of nasopharyngeal carcinoma based on the overlapping mRNAs and lncRNAs. The miRNAs were obtained from differential expression analysis. The network consisted of five lncRNAs, two miRNAs, six mRNAs, and 18 groups of interaction axes (Fig. 3A and Fig. S3A). Among mRNAs, SCARB1 played a significant role in regulating the progression of nasopharyngeal carcinoma. SCARB1 is closely related to the proliferation and invasion of tumor cells, which can enhance tumor adaptability to the microenvironment and inhibit cell apoptosis. So we focused on the ceRNA axis that regulated SCARB1. The results showed that AC010595.1/miR-125b-5p, AC025244.1/miR-125b-5p, LINC01179/miR-125b-5p, LINC02584/miR-125b-5p, and SCAT8/miR-125b-5p axes likely regulated SCARB1 (Fig. 3B).

### SCAT8 may regulate the expression of SCARB1

**Fig. 4:**
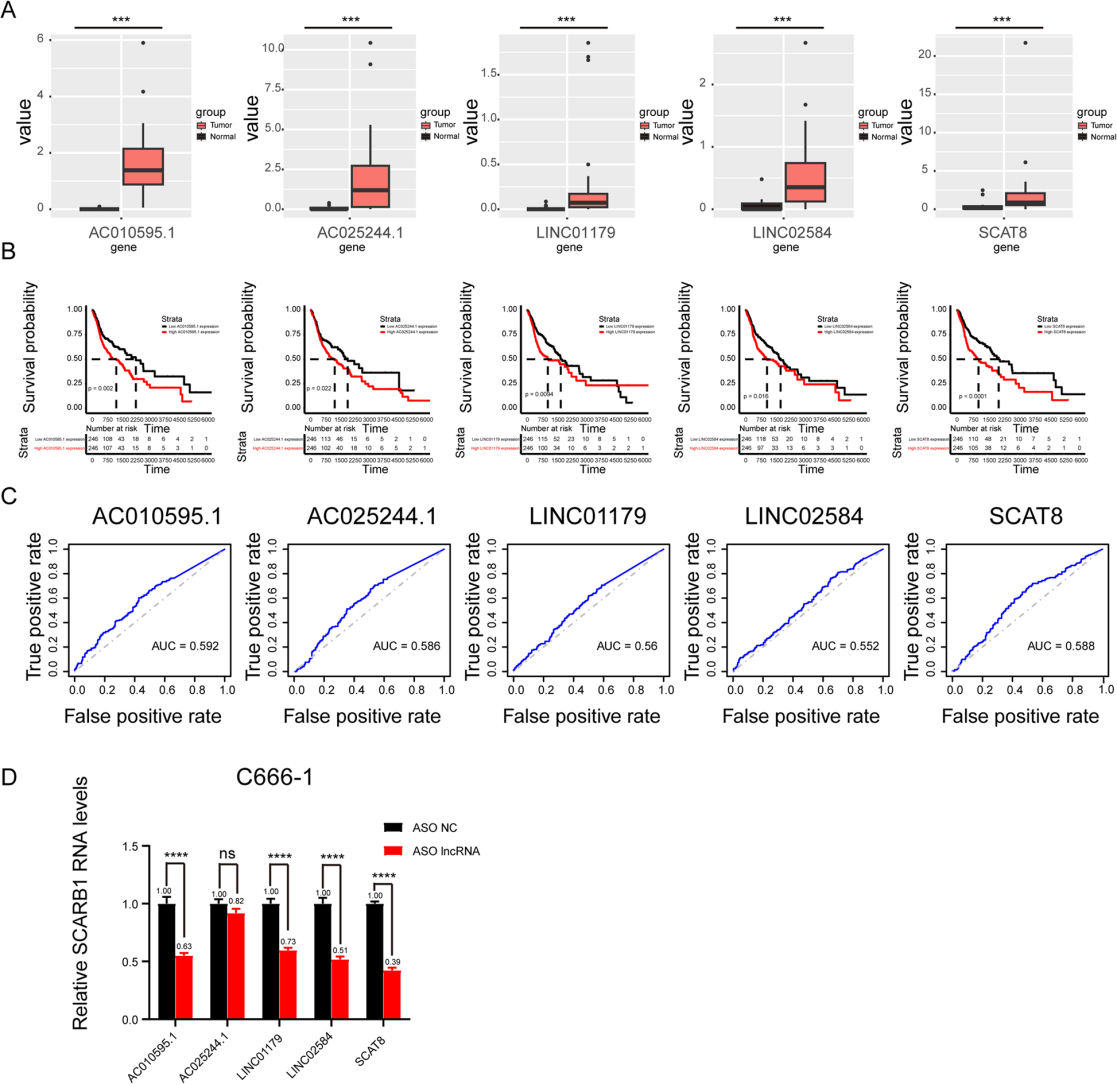
SCAT8 may regulate the mRNA level of SCARB1. The expression of pontential lncRNAs between tumor and normal tissues in nasopharyngeal carcinoma patients. The red box indicates the tumor tissue and the black box indicates the normal tissue (**A**). The survival curves of pontential lncRNAs in nasopharyngeal carcinoma patients. Black lines indicate low expression of lncRNA and red lines indicate high expression of lncRNA (**B**). The ROC curves of the 5 lncRNAs in nasopharyngeal carcinoma (**C**). SCARB1 mRNA levels in C666-1 cells with 5 potential lncRNAs knockdown by ASO (**D**)

To further assess the main lncRNA regulating SCARB1, differential expression analysis and survival analysis were performed for AC010595.1, AC025244.1, LINC01179, LINC02584, and SCAT8. The results of differential expression analysis suggested that five lncRNAs were significantly overexpressed in nasopharyngeal carcinoma (Fig. 4A). Therefore, these lncRNAs could be the reason for the high expression of SCARB1 in nasopharyngeal carcinoma. According to the results of survival analysis and correlation analysis, the lncRNA SCAT8 showed a high correlation with worse survival and the expression SCARB1 (Fig. 4B, Fig. S4A-E). Moreover, the AUC curve showed that the survival sensitivity of SCAT8 was significant (Fig. 4C). To further screen these results, ASO was used to knock down AC010595.1, AC025244.1, LINC01179, LINC02584, and SCAT8 (Fig. 4D). By detecting the changes in the nucleic acid levels of SCARB1, SCAT8 was found to significantly regulate the expression of SCARB1. However, no statistically significant difference was observed when other lncRNAs were knocked down. Thus, SCAT8 may be the main lncRNA regulating SCARB1.

### SCAT8, miR-125b-5p, and SCARB1 regulate the malignant progression of nasopharyngeal carcinoma

**Fig. 5:**
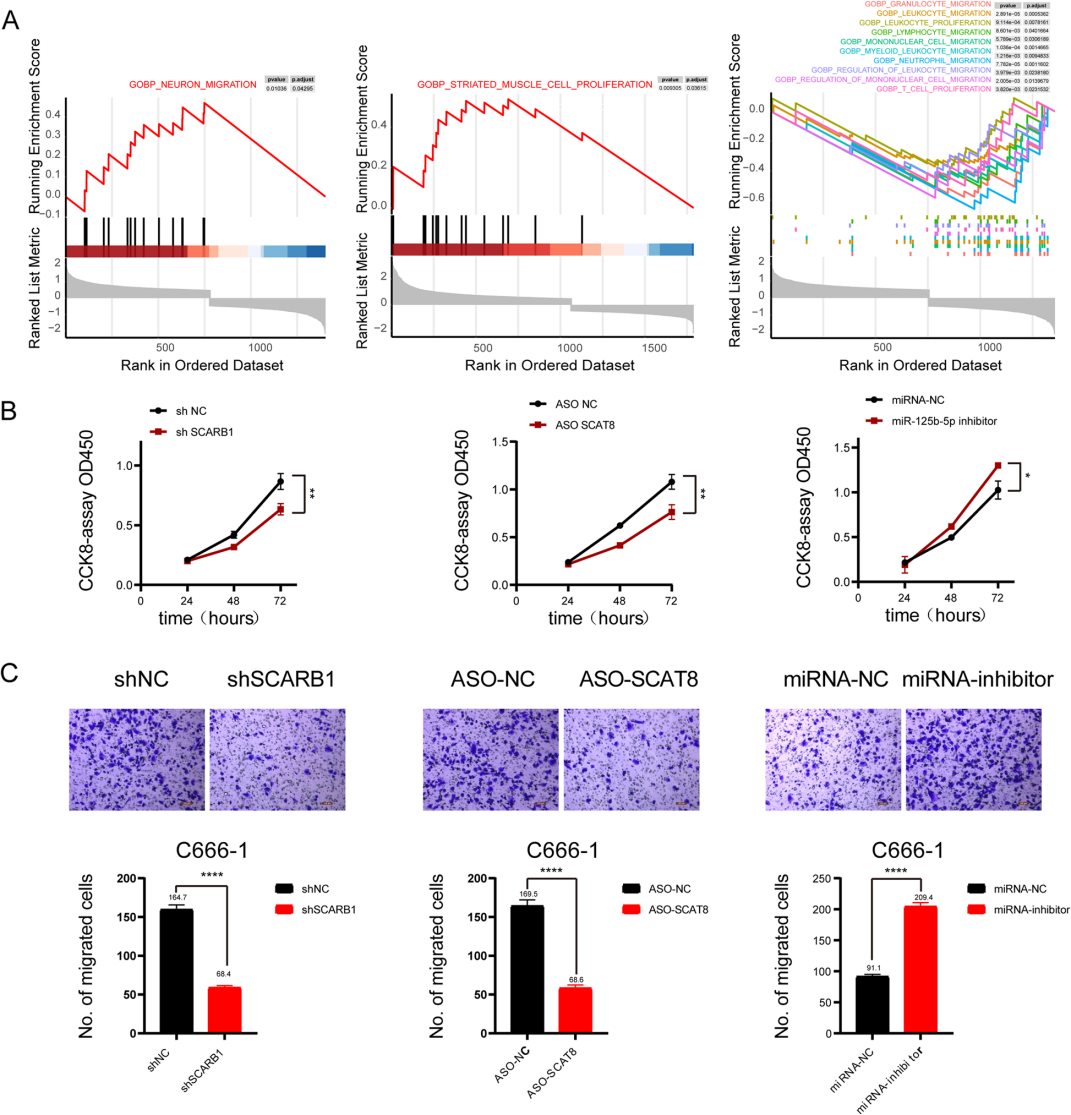
SCAT8, miR-125b-5p and SCARB1 proteins regulate nasopharyngeal carcinoma malignant progression. The C5 GO functional enrichment analysis of the related biological processes of differential expression genes in low expression group and high expression group among SCARB1, SCAT8 and miR-125b-5p (**A**). The CCK8 results in C666-1 cells with SCARB1, SCAT8 and miR-125b-5p knockdown or inhibited (*n* = 3) (**B**). The Transwell results in C666-1 cells with SCARB1, SCAT8 and miR-125b-5p knockdown or inhibited (**C**)

According to the above predictions, SCAT8/miR-125b-5p/SCARB1 may be the ceRNA axis regulating the occurrence and development of nasopharyngeal carcinoma. However, the effect of this ceRNA axis in the malignant progression of nasopharyngeal carcinoma has not been clarified. Therefore, nasopharyngeal carcinoma samples were divided into the SCAT8 high expression group and SCAT8 low expression group for differential expression analysis and enrichment analysis (miR-125b-5p and SCARB1 for the same analysis). The results indicated that the regulatory genes were mainly enriched in cell proliferation and cell migration among other pathways (Fig. 5A and Fig. S5A-C). To further verify the effect of the three genes on the proliferation and migration of nasopharyngeal carcinoma cells, CCK8 and Transwell migration assays were conducted after inhibiting the three genes. These results were consistent with those of enrichment analysis (Fig. 5B, C). The above results indicated that the three genes could separately regulate the malignant progression of nasopharyngeal carcinoma.

### LncRNA SCAT8 and miR-125b-5p can regulate the SCARB1 protein in nasopharyngeal carcinoma

**Fig. 6:**
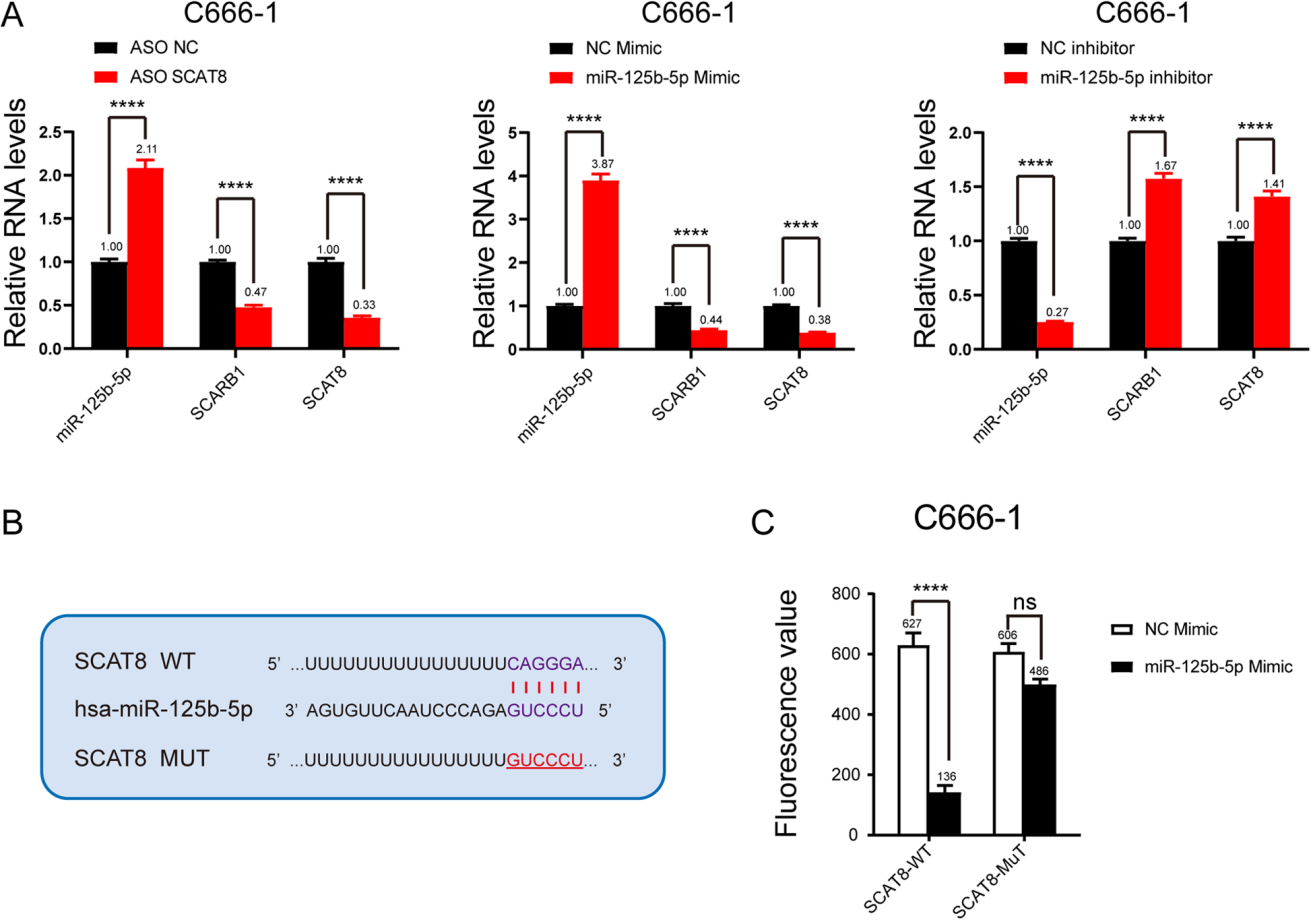
SCAT8 regulates SCARB1 protein and affects the malignant progression of nasopharyngeal carcinoma tumors. RT-qPCR assays were used to detect the mRNA levels of miR-125b-5p, SCARB1 and SCAT8 in C666-1 cell treated with ASO, mimic or inhibitor (*n* = 3) (**A**). Predicted binding site between miR-125b-5p and SCAT8 (**B**). Luciferase assays were performed to test the effect of miR-125b-5p on wild-type or mutant SCAT8 after treating with miR-125b-5p mimic (*n* = 3) (**C**)

To further assess the relationship among SCAT8, miR-125b-5p, and SCARB1, ASO was used to knock down SCAT8. The changes in the levels of miR-125b-5p and SCARB1 expression were detected. The results showed that when SCAT8 was knocked down, the expression of SCARB1 was downregulated, while the expression of miR-125b-5p was upregulated. Next, we detected the changes in SCAT8 and SCARB1-related pathways after overexpression and inhibition of miR-125b-5p with corresponding mimic and inhibitor, respectively. The results showed that SCAT8 and SCARB1-related pathways were negatively regulated by miR-125b-5p (Fig. 6A). In order to explore whether SCAT8 was the ceRNA of miR-125b-5p, the predicted binding sites of SCAT8 and miR-125b-5p were complementarily mutated (Fig. 6B). The reporter gene experiment was used to detect the effect of miR-125b-5p mutation on luciferase activity. Our results suggested that the luciferase activity of wild SCAT8 was regulated by miR-125b-5p, while that of the mutant SCAT8 was not (Fig. 6C). All the above results indicated that the lncRNA SCAT8 and the microRNA miR-125b-5p could regulate the expression of the mRNA SCARB1 in nasopharyngeal carcinoma. These results were consistent with the trend of the ceRNA axis.

### As the ceRNA of miR-125b-5p, SCAT8 regulates SCARB1 to affect the malignant progression of nasopharyngeal carcinoma

**Fig. 7:**
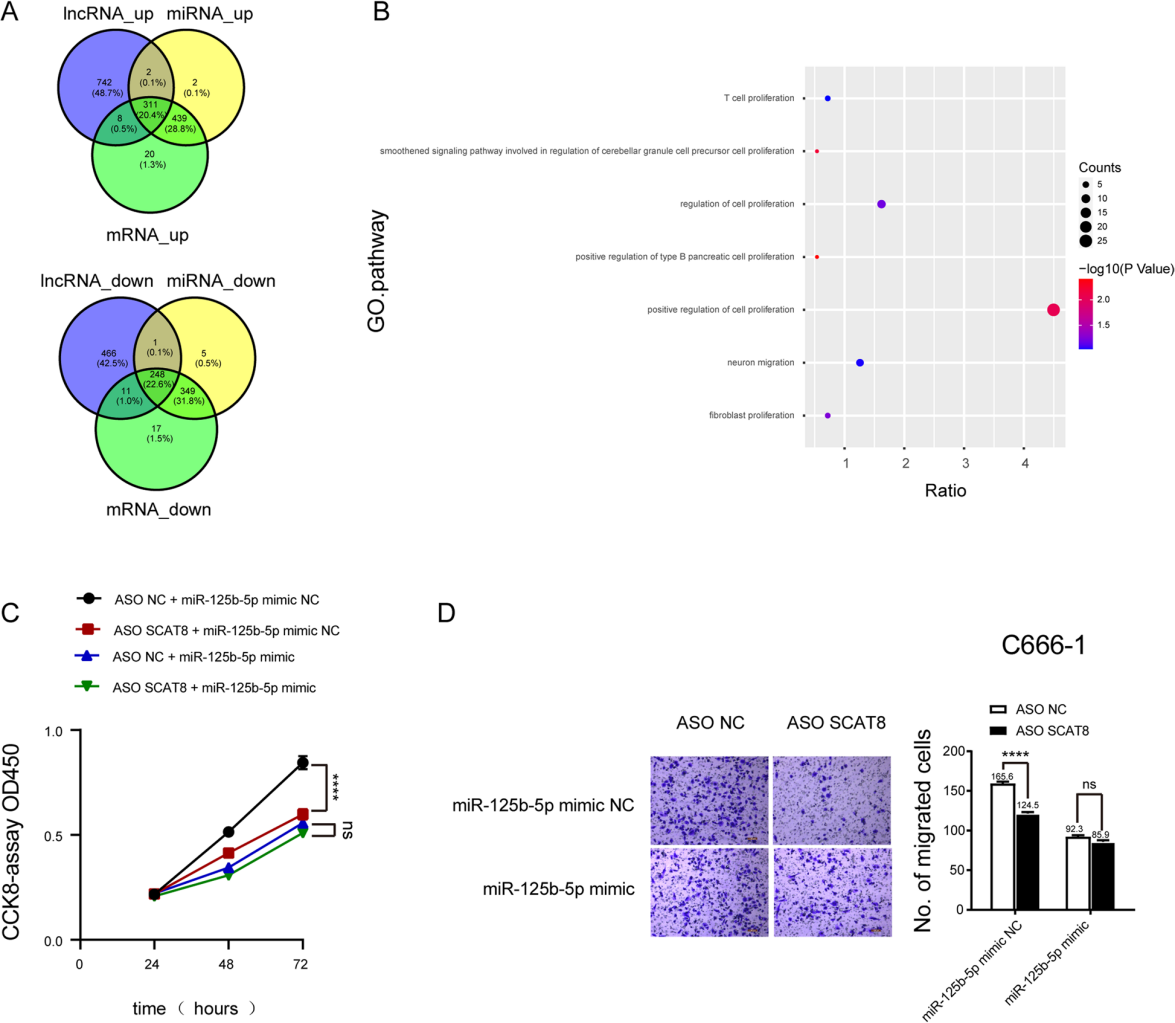
SCAT8 regulates SCARB1 by miR-125b-5p to affect the malignant progression of nasopharyngeal carcinoma. Venn diagrams represent the overlapped genes in low expression group and high expression group among SCAT8, miR-125b-5p, and SCARB1 (**A**). The bubble diagram of GO enrichment analysis of overlapped genes by DAVID database (**B**). The CCK8 results of C666-1 cells with knockdown SCAT8 treated with NC mimic or miR-125b-5p mimic (*n* = 3) (**C**). The Transwell results of C666-1 cells with knockdown SCAT8 treated with NC mimic or miR-125b-5p mimic (**D**)

Next, we predicted the enrichment of two or more genes regulated by the SCAT8/miR-125b-5p/SCARB1 axis in the malignant progression of nasopharyngeal carcinoma. The gene enrichment results showed that the proliferation and migration pathways were particularly significantly overrepresented (Fig. 7A, B and Fig. S6A-D). To verify this finding, SCAT8 was knocked down in the nasopharyngeal carcinoma cell line, C666-1, in control and experimental groups. The experimental group was treated with a miR-125b-5p inhibitor. The proliferation and migration of C666-1 cells were detected by CCK-8 and Transwell migration assays, respectively. The results showed that knocking down SCAT8 inhibited the proliferation and migration of nasopharyngeal carcinoma cells in the control group but this result was no longer observed after using the mimic (Fig. 7C, D). The above results indicated that as the ceRNA of miR-125b-5p, SCAT8 regulated SCARB1 and affected the malignant progression of nasopharyngeal carcinoma.

## Discussion

Nasopharyngeal carcinoma is a highly malignant tumor of the nasopharynx. Due to the obvious early common symptoms, the mortality rate of nasopharyngeal carcinoma remains high. Patients often undergo medical examinations only after they present with obvious symptoms. By this time, more than 70% of patients have progressed to the advanced stage of the disease. Nasopharyngeal carcinoma has the characteristics of high invasiveness, rapid metastasis, poor prognosis, and complex complications [35]. The traditional treatment of nasopharyngeal carcinoma includes surgery, chemotherapy, and radiotherapy but given the deep anatomical site and complex anatomical structure, the curative effect of surgery is limited. Radiotherapy and chemotherapy often lead to decreased immunity and the occurrence of secondary tumors. Therefore, it is significant to develop new and effective treatment methods, enhance the treatment rate and prognosis of patients with nasopharyngeal carcinoma, and effectively reduce their complications. LncRNA is a kind of ncRNA, which exert diverse biological functions. LncRNAs may participate in the occurrence and development of nasopharyngeal carcinoma by interacting with adjacent coding transcripts or proteins. LncRNA can serve as ceRNA and miRNA, thereby regulating each other, participating in the expression of target genes, and playing a key role in tumor processes. Both lncRNAs and miRNAs are involved in the inflammatory cascade of scavenger receptors and subsequent regulation of the lipid metabolism pathways [36]. The expression of SCARB1 in gastric cancer tissues is significantly down-regulated, and its mechanism of action may be associated with the post-transcriptional regulation of SCARB1 mRNA by miRNA [7, 29]. For example, during tumor occurrence, high levels of miR-199 can bind to the 3' UTR of the SCARB1 mRNA, thus inhibiting the expression of SCARB1 [13, 38, 45, 46]. Reduced expression of SCARB1 can induce gastric cancer, which may be related to the regulation of the immune function of CDB + 7 lymphocytes through its expression in the tumor microenvironment. However, the ceRNA network of SCARB1 has not been reported in nasopharyngeal carcinoma. Herein, we found that SCAT8 is a ceRNA of miR-125b-5p that governs the expression of SCARB1 in nasopharyngeal carcinoma. The establishment of a co-regulatory network of SCARB1 and ncRNA in nasopharyngeal carcinoma provides a potential theoretical basis for the diagnosis and treatment of these patients.

SR-BI encoded by the *SCARB1* gene acts as a multifunctional HDL receptor that promotes the uptake of cholesteryl esters by the liver. Recent studies have shown that HDL has excellent properties as an anticancer drug carrier, and the *SCARB1* gene has entered the field of anticancer research [23, 47]. SR-BI is thought to be critical for cholesterol and lipoprotein metabolism in breast and prostate cancer [22, 48]. In all nasopharyngeal carcinoma cell lines and 75% of nasopharyngeal carcinoma biopsies, SR-B1 is overexpressed. Thus, SR-B1 is a potential biomarker for nasopharyngeal carcinoma [38]. The results of the enrichment analysis showed that SCAT8, miR-125b-5p, and SCARB1 could all be involved in cellular proliferation and migration-related pathways, and their regulation by the SCAT8/miR-125b-5p axis was dependent on SCARB1 to a certain extent. Therefore, the SCAT8/ miR-125b-5p/SCARB1 axis may regulate the malignant progression of nasopharyngeal carcinoma through SCARB1.

In conclusion, the regulation of SCARB1 was by modulating the levels of SCAT8 and miR-125b-5p expression. Mechanistically, SCAT8 is a molecular sponge of miR-125b-5p which competitively regulate the expression of an important oncogene, SCARB1, in nasopharyngeal carcinoma. Taken together, the SCAT8/ miR-125b-5p/SCARB1 axis may regulate the malignant progression of nasopharyngeal carcinoma through SCARB1.

## Supplementary Information

**Additional file 1:** **Fig. S1. **The results of univariate cox and differential expression analysis of mRNAs, lncRNAs and miRNAs. PCA diagrams of mRNAs,lncRNAs and miRNAs (A). Venn diagram displaying the intersection genes of the COX analysis and the DEG analysis of mRNAs and lncRNAs (B). **Fig. S2. **C5 GO fuctional and C2 pathway enrichment analysis of the differentally exressed mRNAs. C2 pathway enrichment analysis of regulated mRNAs (A). C5 GO enrichment analysis of regulated mRNAs (B). **Fig. S3. **Construction of ceRNA network by integrated analysis(A). **Fig. S4. **SCAT8 may regulate the mRNA level of SCARB1. Pearson's correlation coefficient between SCARB1 and 5 lncRNAs (AC010595.1, AC025244.1,LINC01179, LINC02584 and SCAT8) (A-E). **Fig. S5. **The differential expression analysis of the SCAT8,miR-125b-5p and SCARB1 in nasopharyngeal carcinoma patients. The volcano plotsand the GSEA of the DEGs in low expression group and high expression groupamong SCARB1 (A), SCAT8 (B) and miR-125b-5p (C). **Fig. S6. **SCAT8 regulates SCARB1 by miR-125b-5p to affect themalignant progression of nasopharyngeal carcinoma. The bubble diagram of GO enrichment analysis of SCAT8 and SCARB1 overlapped genes by DAVID database (A).The bubble diagram of GO enrichment analysis of miR-125b-5p and SCARB1overlapped genes by DAVID database (B). Venn diagrams represent the overlappedgenes in low expression group and high expression group between SCAT8 and SCARB1 (C). Venn diagrams represent the overlapped genes in low expressiongroup and high expression group between miR-125b-5p and SCARB1 (D).

## Data Availability

Bioinformatics data in this manuscript was obtained from the database of TCGA [https://portal.gdc.cancer.gov/].
