# Supplementary material for: SCAT8/miR-125b-5p axis triggers malignant progression of nasopharyngeal carcinoma through SCARB1
Source: BMC Mol Cell Biol. 2023 Apr 3;24:15. doi: 10.1186/s12860-023-00477-2 (PMC10069050; doi:10.1186/s12860-023-00477-2)
Supplement: Supplementary file 1 — Additional file 1: Fig. S1. The results of univariate cox and differential expression analysis of mRNAs, lncRNAs and miRNAs. PCA diagrams of mRNAs,lncRNAs and miRNAs (A). Venn diagram displaying the intersection genes of the COX analysis and the DEG analysis of mRNAs and lncRNAs (B). Fig. S2. C5 GO fuctional and C2 pathway enrichment analysis of the differentally exressed mRNAs. C2 pathway enrichment analysis of regulated mRNAs (A). C5 GO enrichment analysis of regulated mRNAs (B). Fig. S3. Construction of ceRNA network by integrated analysis(A). Fig. S4. SCAT8 may regulate the mRNA level of SCARB1. Pearson's correlation coefficient between SCARB1 and 5 lncRNAs (AC010595.1, AC025244.1,LINC01179, LINC02584 and SCAT8) (A-E). Fig. S5. The differential expression analysis of the SCAT8,miR-125b-5p and SCARB1 in nasopharyngeal carcinoma patients. The volcano plotsand the GSEA of the DEGs in low expression group and high expression groupamong SCARB1 (A), SCAT8 (B) and miR-125b-5p (C). Fig. S6. SCAT8 regulates SCARB1 by miR-125b-5p to affect themalignant progression of nasopharyngeal carcinoma. The bubble diagram of GO enrichment analysis of SCAT8 and SCARB1 overlapped genes by DAVID database (A).The bubble diagram of GO enrichment analysis of miR-125b-5p and SCARB1overlapped genes by DAVID database (B). Venn diagrams represent the overlappedgenes in low expression group and high expression group between SCAT8 and SCARB1 (C). Venn diagrams represent the overlapped genes in low expressiongroup and high expression group between miR-125b-5p and SCARB1 (D). [file 12860_2023_477_MOESM1_ESM.doc]

**Fig. S1**


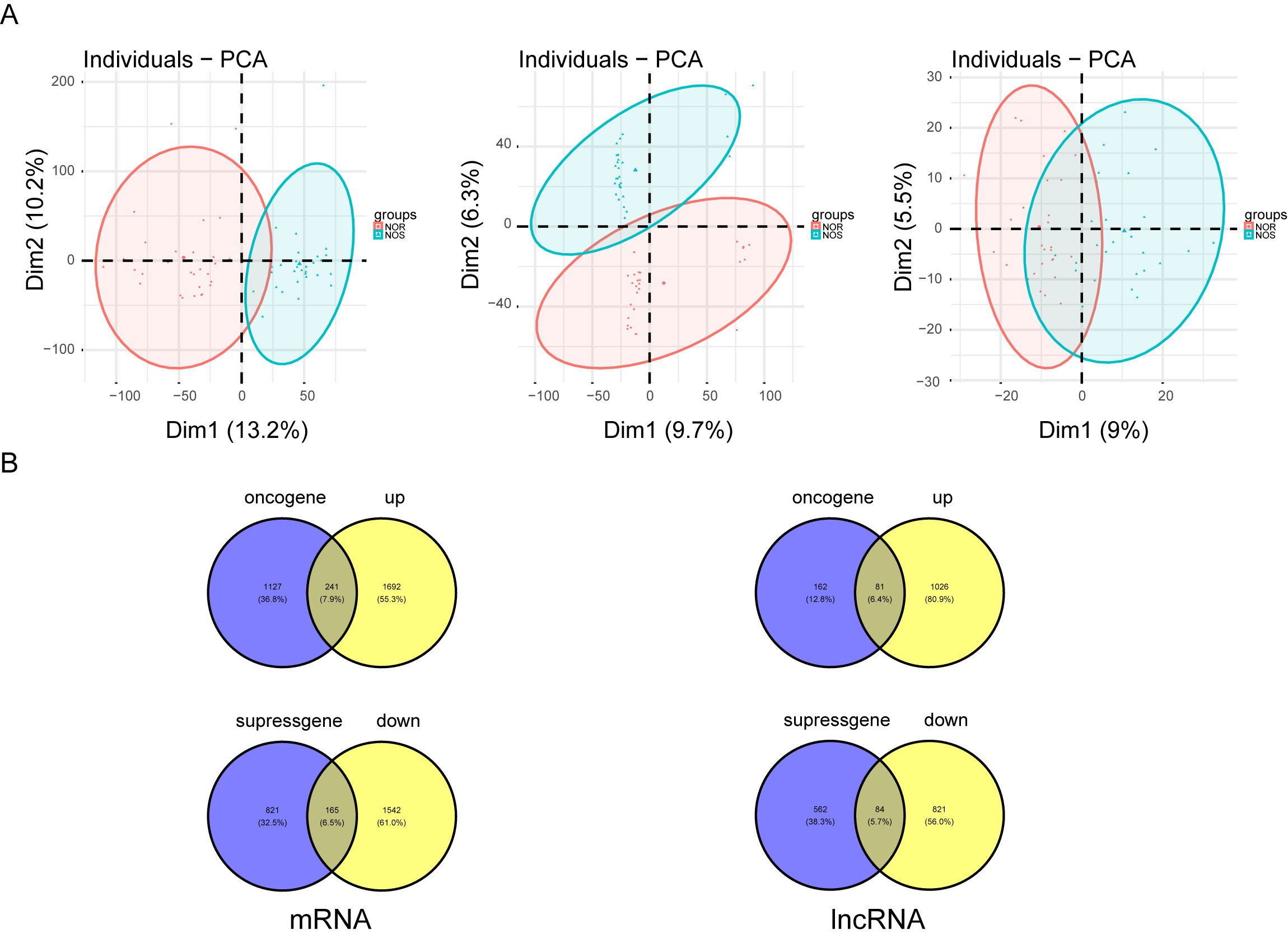


**Fig. S2**


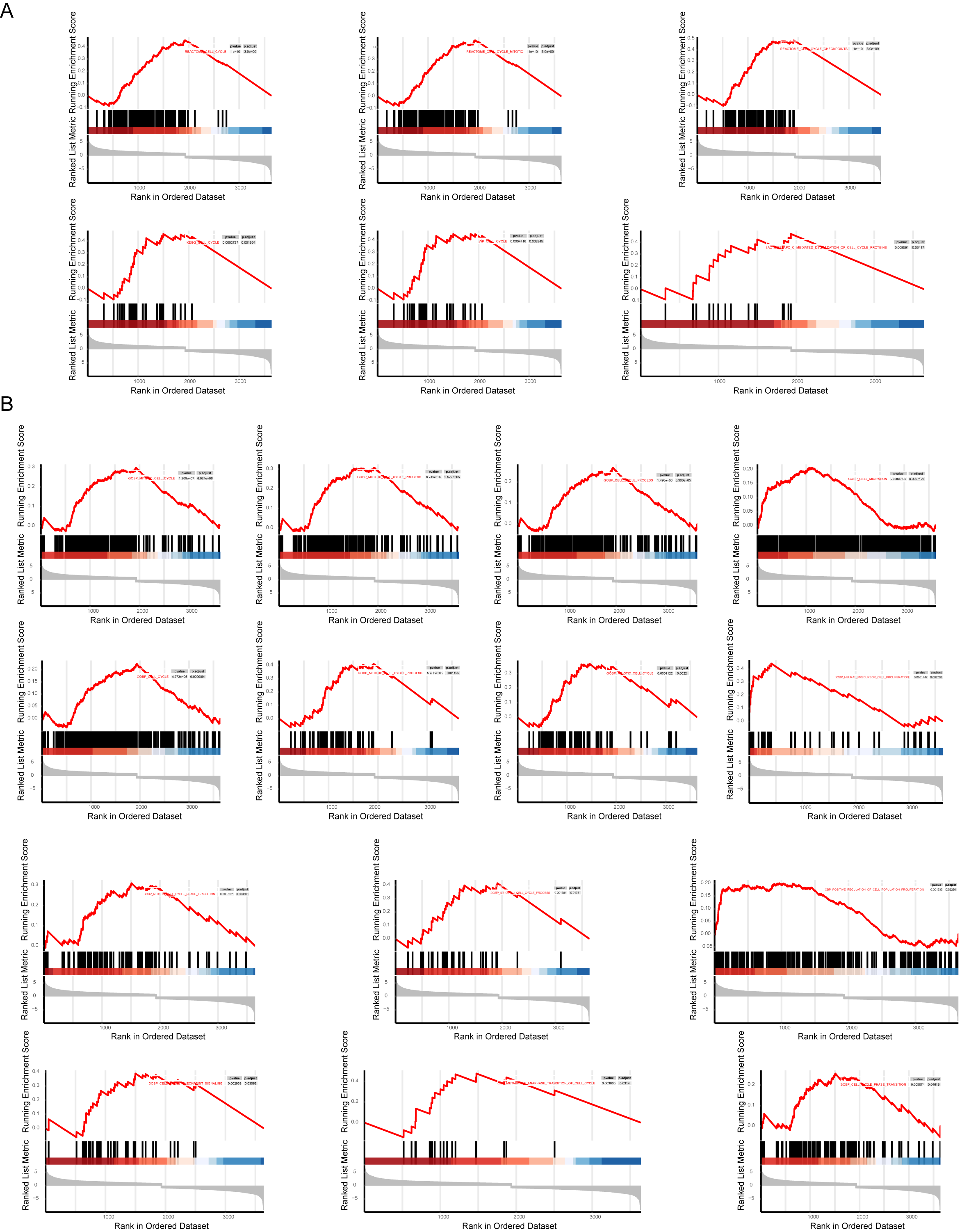


**Fig. S3**


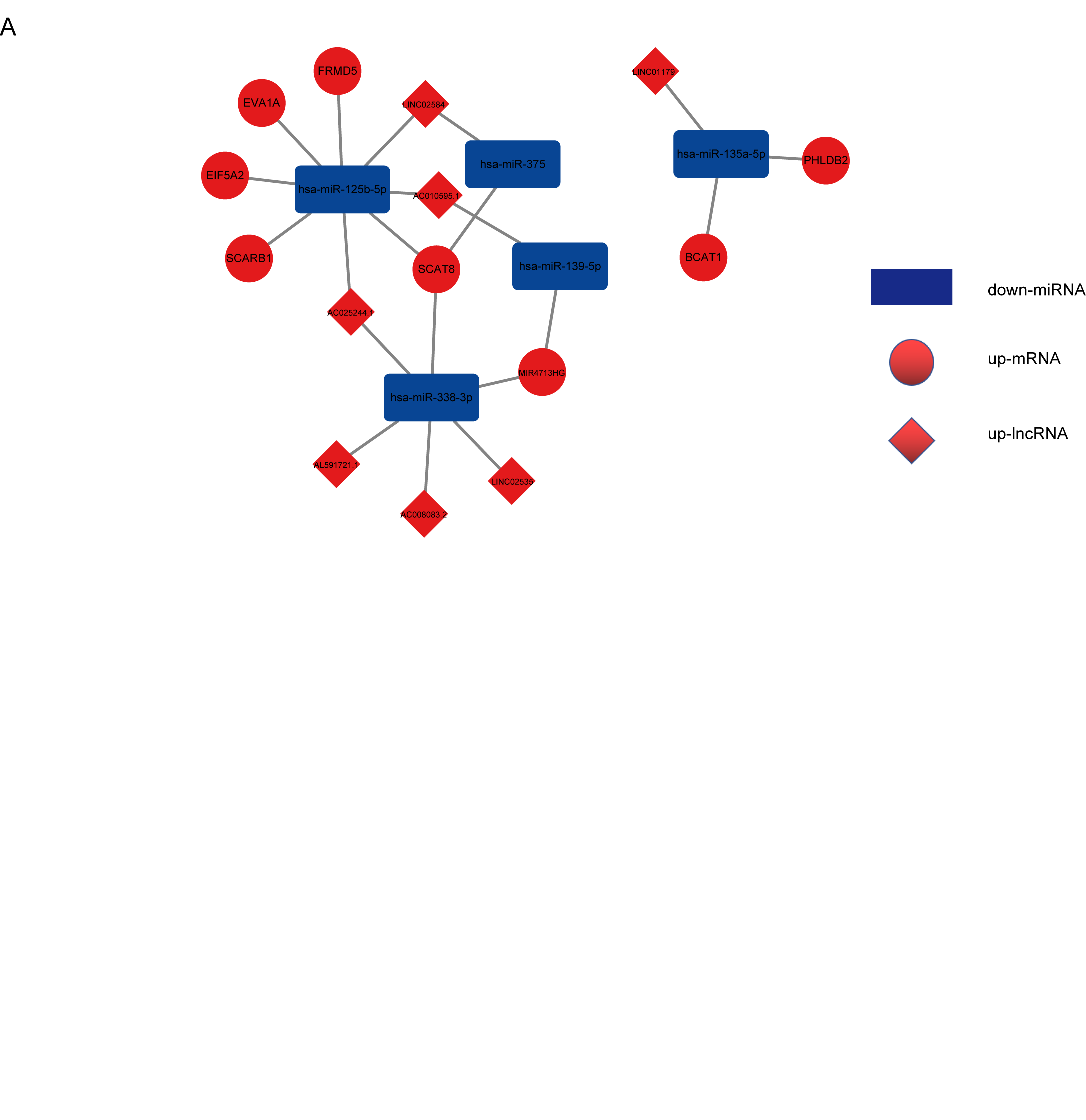


**Fig. S4**

**
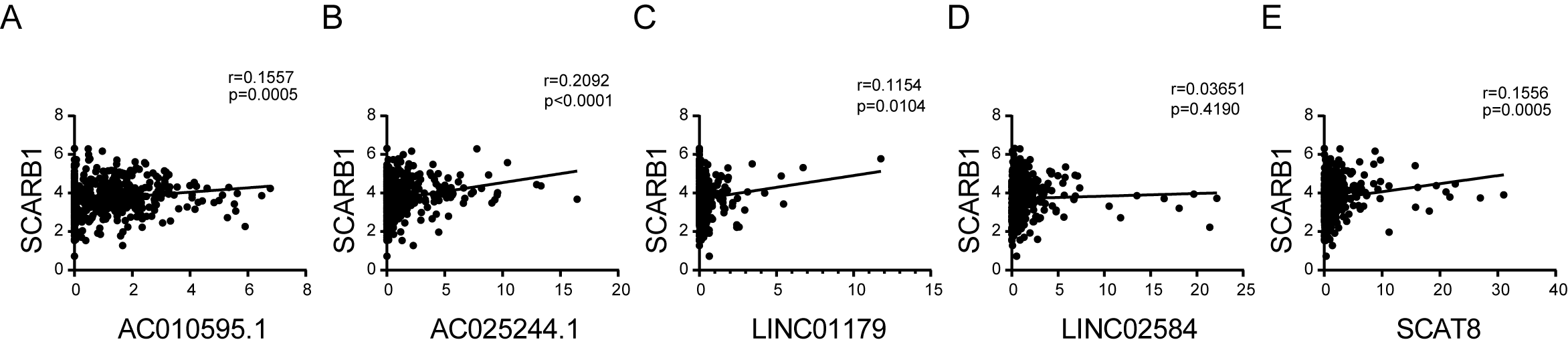
**

**Fig. S5**

**
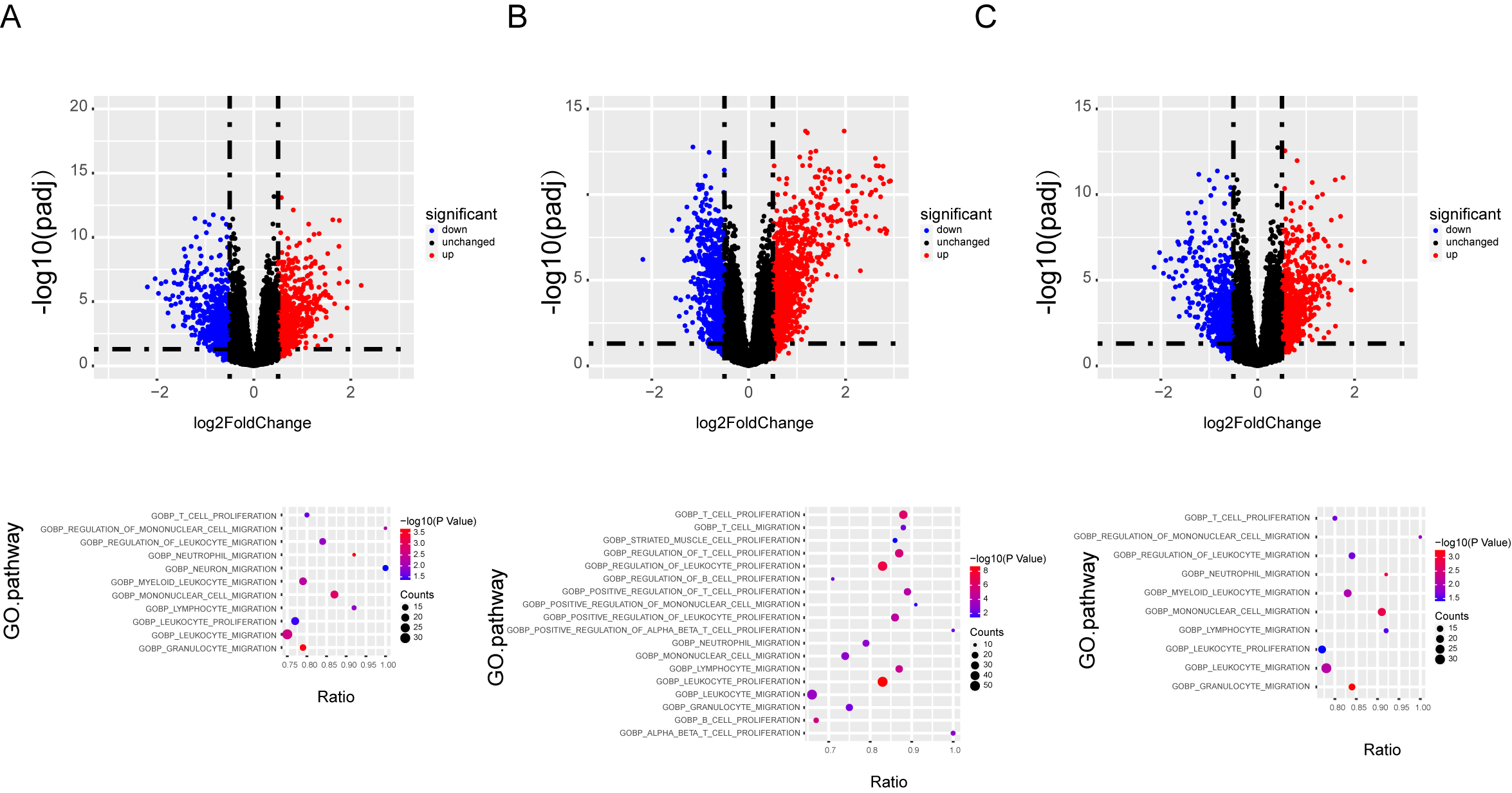
**

**Fig. S6**


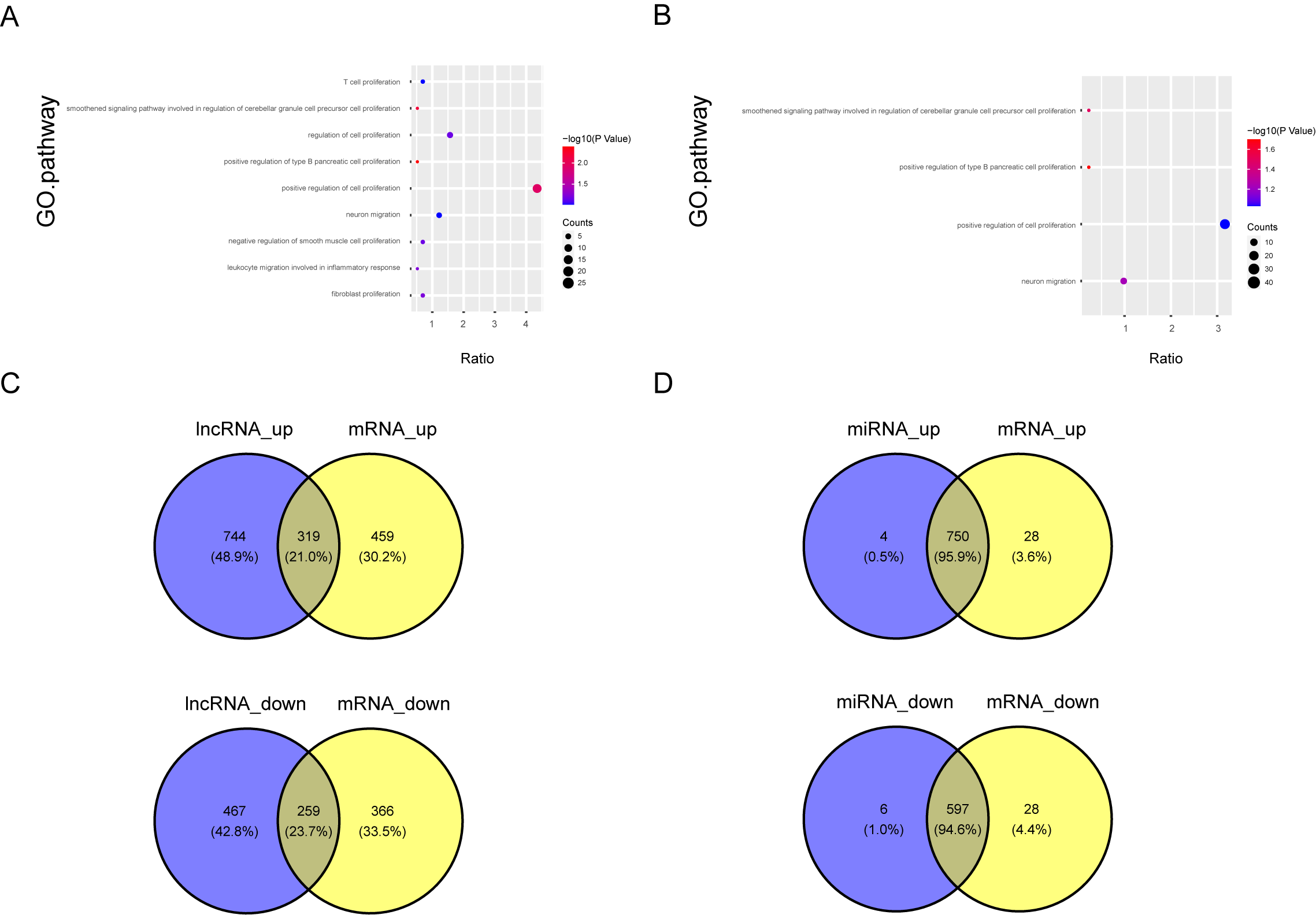


**Fig. S1**

The results of univariate cox and differential expression analysis of mRNAs, lncRNAs and miRNAs. PCA diagrams of mRNAs, lncRNAs and miRNAs (A). Venn diagram displaying the intersection genes of the COX analysis and the DEG analysis of mRNAs and lncRNAs (B).

**Fig. S2**

C5 GO fuctional and C2 pathway enrichment analysis of the diferentally exressed mRNAs. C2 pathway enrichment analysis of regulated mRNAs (A). C5 GO enrichment analysis of regulated mRNAs (B).

**Fig. S3**

Construction of ceRNA network by integrated analysis (A).

**Fig. S4**

SCAT8 may regulate the mRNA level of SCARB1. Pearson's correlation coefficient between SCARB1 and 5 lncRNAs (AC010595.1, AC025244.1, LINC01179, LINC02584 and SCAT8) (A-E).

**Fig. S5**

The differential expression analysis of the SCAT8, miR-125b-5p and SCARB1 in nasopharyngeal carcinoma patients. The volcano plots and the GSEA of the DEGs in low expression group and high expression group among SCARB1 (A), SCAT8 (B) and miR-125b-5p (C).

**Fig. S6**

SCAT8 regulates SCARB1 by miR-125b-5p to affect the malignant progression of nasopharyngeal carcinoma. The bubble diagram of GO enrichment analysis of SCAT8 and SCARB1 overlapped genes by DAVID database (A). The bubble diagram of GO enrichment analysis of miR-125b-5p and SCARB1 overlapped genes by DAVID database (B). Venn diagrams represent the overlapped genes in low expression group and high expression group between SCAT8 and SCARB1 (C). Venn diagrams represent the overlapped genes in low expression group and high expression group between miR-125b-5p and SCARB1 (D).
